# Advancing socioecological mental health promotion intervention: A mixed methods exploration of Phase 1 Agenda Gap findings

**DOI:** 10.3389/fpubh.2023.1066440

**Published:** 2023-02-16

**Authors:** Emily Jenkins, Zachary Daly, Corey McAuliffe, Liza McGuinness, Chris Richardson, Taylor G. Hill, Trevor Goodyear, Candace Lind, Skye Barbic, Robert Rivers, Rebecca Haines-Saah

**Affiliations:** ^1^School of Nursing, University of British Columbia, Vancouver, BC, Canada; ^2^School of Population and Public Health, University of British Columbia, Vancouver, BC, Canada; ^3^Department of Psychology and Neuroscience, Dalhousie University, Halifax, NS, Canada; ^4^Faculty of Nursing, University of Calgary, Calgary, AB, Canada; ^5^Department of Occupational Science and Occupational Therapy, University of British Columbia, Vancouver, BC, Canada; ^6^Foundry, Vancouver, BC, Canada; ^7^Health Policy Consultant, Vancouver, BC, Canada; ^8^Cumming School of Medicine, University of Calgary, Calgary, AB, Canada

**Keywords:** youth, mental health, mental health promotion, advocacy, policy, intervention, mixed methods, community-based research

## Abstract

**Introduction:**

Protecting and promoting the mental health of youth under 30 years of age is a priority, globally. Yet investment in mental health promotion, which seeks to strengthen the determinants of positive mental health and wellbeing, remains limited relative to prevention, treatment, and recovery. The aim of this paper is to contribute empirical evidence to guide innovation in youth mental health promotion, detailing the early outcomes of Agenda Gap, an intervention centering youth-led policy advocacy to influence positive mental health for individuals, families, communities and society.

**Methods:**

Leveraging a convergent mixed methods design, this study draws on data from n = 18 youth (ages 15 to 17) in British Columbia, Canada, who contributed to pre- and post-intervention surveys and post-intervention qualitative interviews following their participation in Agenda Gap from 2020-2021. These data are supplemented by qualitative interviews with n = 4 policy and other adult allies. Quantitative and qualitative data were analyzed in parallel, using descriptive statistics and reflexive thematic analysis, and then merged for interpretation.

**Results:**

Quantitative findings suggest Agenda Gap contributes to improvements in mental health promotion literacy as well as several core positive mental health constructs, such as peer and adult attachment and critical consciousness. However, these findings also point to the need for further scale development, as many of the available measures lack sensitivity to change and are unable to distinguish between higher and lower levels of the underlying construct. Qualitative findings provided nuanced insights into the shifts that resulted from Agenda Gap at the individual, family, and community level, including reconceptualization of mental health, expanded social awareness and agency, and increased capacity for influencing systems change to promote positive mental health and wellbeing.

**Discussion:**

Together, these findings illustrate the promise and utility of mental health promotion for generating positive mental health impacts across socioecological domains. Using Agenda Gap as an exemplar, this study underscores that mental health promotion programming can contribute to gains in positive mental health for individual intervention participants whilst also enhancing collective capacity to advance mental health and equity, particularly through policy advocacy and responsive action on the social and structural determinants of mental health.

## Introduction

The mental health of youth under 30 years of age has long been a public health priority. Mental health challenges are among the leading causes of health- and disability-related burden globally (1); with most mental illnesses first arising in adolescence (2). While population mental health represents a clear target for public health action, contributors to positive mental health and wellbeing – the domain of mental health promotion – are often overlooked in research, policy, and practice (3, 4). Mental health promotion is a strengths-based orientation to advancing positive mental health and equity by building individual and community capacity to identify and redress relevant barriers (5). By operating “upstream,” mental health promotion targets the social determinants of mental health, or the everyday circumstances and social and structural forces that shape opportunities for health and wellbeing. As such, mental health promotion holds the potential to impact mental health across socioecological domains, meaning that it can be designed to strengthen positive mental health at the individual (e.g., health status, coping), family (e.g., relationships, income), community (e.g., social cohesion, built environment), and societal levels (e.g., discrimination, equity) (6).

In the youth mental health sphere, mental health promotion has been implemented in a number of settings, including in schools (7, 8), online (9), and in community-based settings (10, 11). Often, this work has focused on reaching priority population groups, such as LGBTQ2+ youth (12) and urban youth living in underserved communities (13). The aims of mental health promotion interventions have ranged from building personal skills and competencies, such as coping, stress management, and self-efficacy (7); through to strengthening youth-adult relationships, empowering youth through civic engagement and shared decision making (11), and shifting community norms and practices related to how youth are prioritized in society (10).

Yet, despite its promise, mental health promotion has received a disproportionately low level of attention and investment compared to its illness-oriented counterparts – that is, prevention, treatment, and recovery (3). This has resulted in more limited and pilot-based programming and a stunted evidence base to guide intervention and funding. Indeed, in a review of youth mental health promotion programs in schools, O'Mara and Lind (8) concluded that “study populations are limited and many studies either lack clarity regarding who implemented interventions, lack theoretical foundations, process evaluations or youth viewpoints” (p. 203). Moreover, in a review of reviews conducted by Enns et al. (14), it was found that the scope of mental health promotion intervention remains predominantly focused on individual protective factors, with much less attention to interventions intended to alter the broader social and structural determinants of mental health and wellbeing for communities or populations. We argue that this reflects a missed opportunity and fails to acknowledge, as Mantoura (6) writes, that “[i]mproving mental health is social and political; it requires interventions in all sectors and settings people traverse during their life trajectory” (p. 15). Addressing the “social” and “political” requires a range of approaches and tools, including policy advocacy, which has been identified as a key mental health promotion strategy. Given the gaps and opportunities identified, the purpose of this paper is to advance the empirical evidence base for youth mental health promotion through an exploration of the early Phase 1 findings of Agenda gap – an innovative mental health promotion intervention that centers youth-led policy advocacy. Data collected during Phase 1, and presented here, will be used to inform the expansion of Agenda Gap in Phase 2 to additional study sites, alongside ongoing evaluation.

## Materials and methods

### Intervention overview

#### Guiding intervention theories

Agenda Gap is a social innovation supported by the Public Health Agency of Canada's Mental Health Promotion Innovation Fund (MHP-IF), which provided Phase 1 (2019–2022) funding and recently awarded Phase 2 funding (2022–2026). The MHP-IF “funds the testing and delivery of promising population health interventions in the area of mental health promotion with an emphasis on increasing health equity”, including through addressing “systemic barriers for population mental health in Canada” (15). During Phase 1, the focus was on the initial development and delivery of the Agenda Gap intervention.

The Agenda Gap intervention was developed through partnerships with youth from diverse groups and/or backgrounds in the lower mainland of British Columbia, Canada. Aligned with the goals and values of health promotion generally (16) and the MHP-IF more specifically, it is guided by the theoretical tenets of mental health promotion, positive youth development, community youth development, and liberation psychology. Specifically, the overarching framework for Agenda Gap is mental health promotion theory, which directs a focus on positive mental health, as opposed to mental ill health. It further informs the intervention focus on policy as a strategy for strengthening positive mental health across socioecological domains by enhancing conditions conducive to wellbeing (5, 17). Positive Youth Development encourages meaningful youth engagement to foster progression in developmental competencies, particularly among youth who are marginalized, while Community Youth Development brings focus to issues of social justice and equity as well as community- or population-level impacts. Finally, the Theory of Sociopolitical Development (18), which originates from the traditions of liberation psychology, drives an intervention that is responsive to the root determinants of mental health and builds “capacity to identify, analyze, and act on issues relevant to youth” (19). Together, these theories guide an intervention that centers a human rights approach to action on the social determinants of health and equity to strengthen mental health and wellbeing for individuals and their communities.

#### Intervention implementation

Agenda Gap centers youth expertise and prepares youth collaborators for meaningful policy engagement (i.e., multi-level and multi-sectoral action and advocacy for systems change) to promote mental health of individuals, families, communities, and society. In October 2020, Agenda Gap launched in its first two intervention sites in British Columbia, followed by an additional intervention site in Alberta in October 2021. Forthcoming sites in the provinces of Ontario and Nova Scotia are set to launch in 2023 as part of Phase 2 activities. To date, Agenda Gap has been delivered entirely online due to the COVID-19 pandemic and associated public health protections. The intervention consists of: (1) a youth mental health promotion and policy advocacy “curriculum” delivered through a developmental relationship building process, (2) facilitator and ally (community, policy and other decision maker) capacity-generating activities, and (3) strategic knowledge mobilization.

Youth collaborators (aged 15–24 years) are recruited through partner organization networks, including schools, community organizations and health services, with an emphasis on engaging youth who experience intersecting health and social inequities (e.g., have accessed mental health services, live in poverty, are in care of the child welfare system, or who are racialized, Indigenous and/or LGBTQ2+). Interested youth are interviewed to explore interest and to curate cohorts of 5–15 youth with shared experiences or passions. Cohorts are then engaged *via* weekly 2-hour facilitated sessions over ~6 months, with youth financially compensated through an hourly honorarium. Facilitators, identified through partner organizations,are mentored through the implementation process by the research team through a train-the-trainer process. This is further supported by a Facilitation Guide detailing activities to promote skills building and collective policy advocacy beyond the conclusion of the formal intervention. Core session topics include: mental health promotion literacy, social and structural determinants of mental health and (in)equity, youth rights as a platform for policy advocacy, and influencing systems and system actors [see Jenkins et al. (20) for further details on intervention content and protocol]. Materials to equip policy and other decision makers to support meaningful youth engagement in policy making are also disseminated to adult allies engaging with youth during the intervention, while multi-pronged knowledge mobilization strategies are leveraged to broaden and deepen impacts beyond intervention participants (e.g., media interviews; school, community, and conference presentations; infographics; policy briefings; collaborative policymaking). The overarching aim of these intervention processes are to equip youth collaborators to:

1) Collectively identify factors in their community that impact youth mental health *and* are amenable to change through policy.
2) Develop strategies and action plans to effect relevant policy development/change, including through knowledge mobilization outputs.
3) Engage with relevant parties, including policymakers, in collaborative policymaking processes to promote youth mental health.

In this way, Agenda Gap is designed to contribute to impacts across the four socioecological domains. Youth participants benefit directly through the development of supportive relationships and sense of connectedness, as well as new skills and knowledge about the links between mental health and policy (individual level). Moreover, policy and other adult allies are engaged and leveraged through collaborative policy-making processes with youth (individual and community levels). Together, this serves to advance mental health promotion and equity for – and with – youth and others living in communities where the policy advocacy and intervention is targeted (family, community and societal levels) (21).

### Study design and conceptual framework

**Figure 1:**
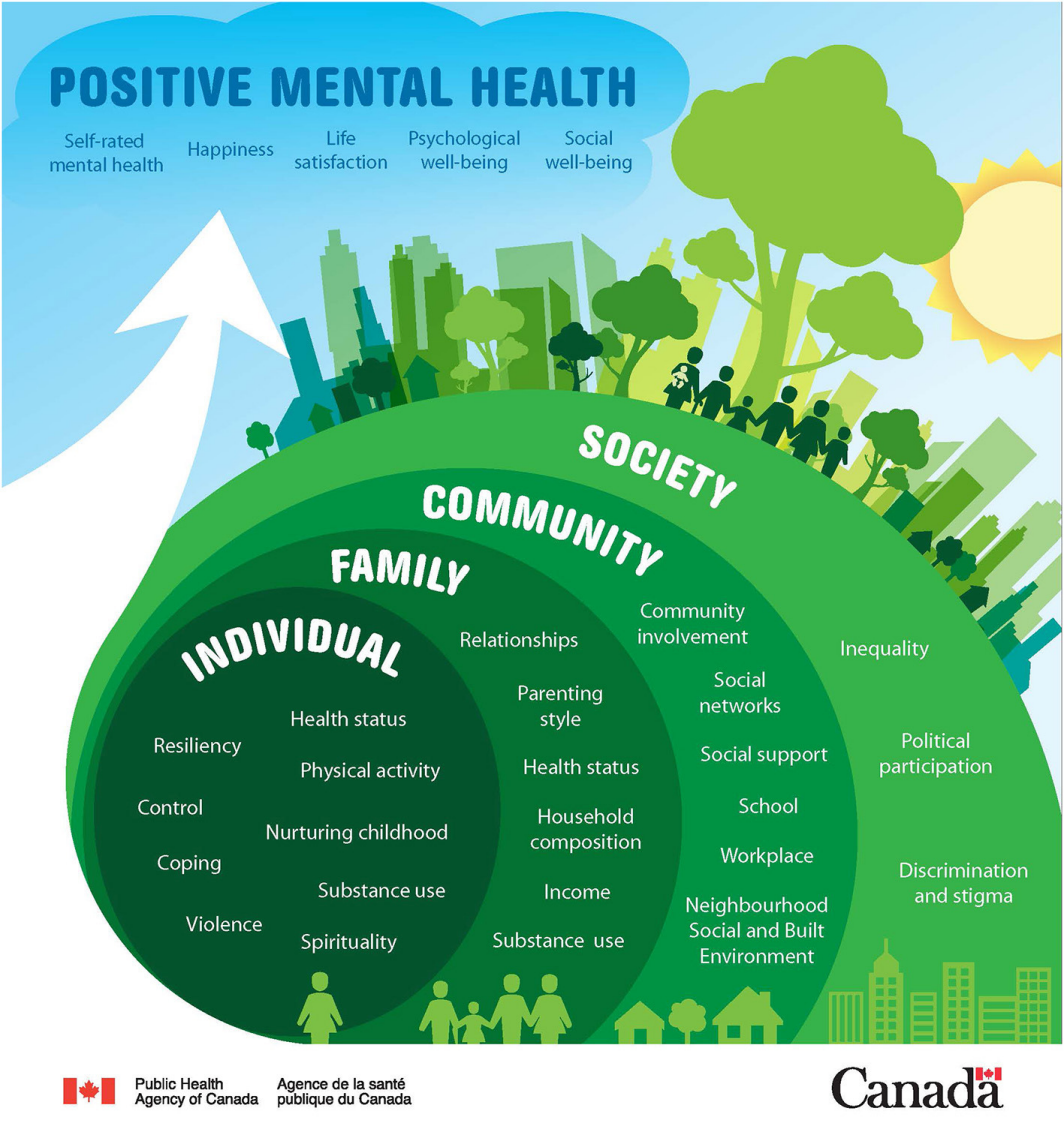
Positive mental health conceptual framework for surveillance. Developed by Orpana et al. (21).

A convergent mixed-methods design (22) guided by realist evaluation methodology (23) is utilized alongside the intervention to allow for exploration into *how* Agenda Gap works, for *whom* and in what *contexts*. It also provides data that can be used to investigate youth and adult ally perspectives and measures of intervention impact, which is the focus of the present paper. Conceptually, this exploration is guided by the Positive Mental Health Surveillance Indicator Framework, developed by the Public Health Agency of Canada (21) (see Figure 1).

Aligned with mental health promotion theory, this framework adopts a socioecological orientation to positive mental health and identifies risk and protective factors across individual, family, community, and societal domains. Utilizing this framework orients an investigation of Agenda Gap impacts for individuals as well as “ripple effects” that reach beyond those directly involved in the intervention (24).

### Data collection

The overarching study comprises a variety of data sources, including anonymous pre- and post-intervention online surveys, pre-intervention qualitative interviews, post-intervention qualitative interviews, research logs, and impact mapping. This paper draws on data from the anonymous pre- and post-intervention online surveys and the post-intervention qualitative interviews. These were collected with Phase 1 youth collaborators from the first two intervention cohorts in British Columbia. Data were collected between September 2020 (pre-intervention survey) and June 2021 (post-intervention survey and interviews). Additional data comes from post-intervention qualitative interviews, conducted in June and July 2021 with a subset of policy/decision makers who were engaged in intervention activities with these cohorts. The two youth cohorts, from which the Phase 1 data used in the present analysis were drawn from, were recruited through partner organizations in the recreation and education sectors and included those living in an urban neighborhood that is characterized by high levels of poverty and other health and social inequities, as well as youth living in a suburban neighborhood that is home to a high proportion of new immigrant families. In addition to receiving an hourly honoraria for participation in the Agenda Gap intervention, all youth received $20 CAD for each survey or interview that they participated in to acknowledge their time and contributions to the study. Adult participants were not financially compensated for their time as their participation was considered within the scope of their professional role. Ethical approval for the study was obtained by the University of British Columbia Behavioral Research Ethics Board (H17-001602). Informed consent was provided upon initiating the online survey and orally prior to the beginning of each interview.

### Quantitative measures of positive mental health

Anonymous pre- and post-intervention online surveys drew on a number of measures designed to assess constructs of positive mental health, as guided by our intervention theories (see Table 1 for overview of intervention theories mapped to corresponding socioecological domains and measures). Their use in this study also served as an opportunity to determine their utility for subsequent phases of research, including program evaluation. A knowledge assessment was also constructed by our research team to measure changes in what we conceptualize as mental health promotion literacy, or individuals' knowledge and beliefs about the determinants of positive mental health and wellbeing. Surveys were administered to participants *via* Qualtrics.

**Table 1:** Intervention theories mapped to corresponding socioecological domains and measures.

**Theory**	**Measures**	**Socioecological domains represented**
Mental Health Promotion Socio-ecological orientation to positive mental health, actions to alter the social and structural determinants of mental health, including through policy advocacy	CYRM-12: A Brief Measure of Resilience	Individual, Family, Community
	Peer and Adult Relationships	Individual, Family, Community
	Knowledge assessment – mental health promotion literacy	Individual, Family, Community, Society
Positive Youth Development (PYD) Growth in developmental competencies	General Self-Efficacy Scale (GSE)	Individual
Community Youth Development (CYD) Capacity to identify and redress social inequities Liberation psychology Empowerment, and the capacity to identify, analyze, and act on issues relevant to youth	Individual, Family, Community, Society	Critical Consciousness Scale Civic Participation

#### Knowledge

A series of 10 questions were developed by our research team to assess participant knowledge across intervention time points. Questions reflect key concepts related to positive mental health, including mental health promotion, youth rights, and the relationship between mental health and policy.

#### General self-efficacy scale

The 10-item General Self-Efficacy Scale (25) was adopted for this study. This scale measures participants' perceived competence in responding to stressful circumstances. Responses to the items comprising this scale were measured on a 4-point Likert scale ranging from 1 (Not at all true) to 4 (Exactly true). A sample item is, “I can usually handle whatever comes my way.” In a multicultural validation study, this measure was found to have acceptable reliability levels in community-based samples, including among students (α = 0.86 to 0.90) (26).

#### CYRM-12: A brief measure of resilience

The 12-item Child and Youth Resilience Measure (CYRM) (25) was used as a brief, multidimensional measure of resilience in young people. Participants' responses to the items comprising this scale were measured on a 7-point Likert scale from 1 (Does not describe me at all) to 7 (Describes me a lot). A sample item is, “I know where to go in my community to get help” and the original validation of the scale showed acceptable reliability (α = 0.84) (27).

#### Critical consciousness scale

The Critical Consciousness Scale (CCS) is a 22-item scale comprised of three subscales: Critical Reflection: Perceived Inequality (items 1–8); Critical Reflection: Egalitarianism (items 9–13); and Critical Action: Sociopolitical Participation (items 14–22). The scale was developed and tested among diverse youth populations to quantify critical consciousness, conceptualized as the capacity of marginalized peoples to critically analyze “their social conditions and individual or collective action taken to change perceived inequities” (28). In this study, we utilized the Perceived Inequality and Egalitarianism subscales (items 1–13). Participants' responses to the items comprising this scale were measured on a 6-point Likert scale ranging from 1 (Strongly Disagree) to 6 (Strongly Agree). For the eight-item Perceived Inequality subscale, a sample item is, “Certain racial or ethnic groups have fewer chances to get ahead”. This subscale showed moderate internal consistency in the original validation (α = 0.84). For the five-item Egalitarianism subscale, a sample item is, “It would be good if groups could be equal”. This subscale also demonstrated acceptable internal consistency in the original validation (α = 0.82) (29).

#### Peer and adult attachment

Both peer and adult attachment were assessed through measures adapted from various sources by the Students Commission of Canada for their Youth and Community Survey (30). Specifically, the peer attachment questions draw from the research of Armsden and Greenberg (31), which utilized attachment theory principles to develop a measure of youth's feelings about their significant attachment relationships, including with peers. Similarly, the questions assessing adult attachment were adapted by the Students Commission of Canada from the work of Whitlock (32), which focused on community connectedness, including relationships between youth and adults. Peer attachment was measured through three-items, including: “My friends are there when I need them” on a 5-point scale ranging from 1 (Never true) to 5 (Always true). Adult attachment was measured through five-items, including “There are adults I can ask for help when I need it” on a 5-point scale ranging from 1 (Strongly disagree) to 5 (Strongly agree). While there is no published literature exploring the validity of these measures, they hold strong face validity and were considered well aligned with our guiding intervention theories.

#### Civic participation

The 10-item Civic Participation Scale was adopted from the Youth and Community Survey developed by the Students Commission of Canada (30). The Civic Participation Scale draws on the theoretical and research-based contributions of Pancer et al. (33), Speers and Peterson (34), and Flanagan et al. (35), all of which center the developmental importance of youth involvement in social and political aspects of life. Participants were asked about the frequency of their behaviors, such as involvement in community activities, including volunteering, as well as political activities such as taking part in discussions about social or political issues over the past year. Participants answered on a 5-point Likert scale ranging from 1 (Never) to 5 (A lot), reflecting how often they participated in each activity. While there is no published literature exploring the validity of this measure, it also has strong face validity and was considered well aligned with our guiding intervention theories.

### Qualitative interviews

Qualitative interviews were guided by a semi-structured interview guide. This supported detailed accounts of participant experiences and perspectives related to the intervention and its effects. Questions included a focus on perceived intervention impacts for participants (e.g., *We expect that the Agenda Gap program will improve policies for youth mental health. That's one outcome, but we are also interested in the immediate positive or negative effects of being involved. Can you describe any positive impacts to you personally? Can you describe any negative impacts to you personally?*), as well as impacts within participants' broader communities (e.g., *Now that you have participated in Agenda Gap, in what ways have you seen things shift (outlook, relationships) among your peers because of your participation? What shifts have you noticed in your school, at home or in your community, if any?*). To enhance participant comfort in sharing all aspects of their intervention experience, interviews were conducted *via* Zoom and audio recorded by two members of the study team who were not involved in intervention implementation. Recordings were uploaded to Temi, an automated transcription service, and then checked for accuracy.

### Data analysis

All pre- and post-intervention survey data were analyzed using SPSS 26 to produce descriptive statistics to characterize demographics, knowledge, and measures of positive mental health pre- and post-intervention. Paired sample *t*-tests were conducted to assess change over time on positive mental health assessments. This process facilitates the trialing of selected measures for consideration of their fit with Agenda Gap constructs for use in Phase 2.

All qualitative interview transcripts were de-identified and uploaded to NVivo 12 to facilitate coding. Reflexive thematic analysis techniques were used as an initial analytic tool to examine and interpret the qualitative interview data and construct key intervention impacts from the perspectives of youth and adult allies (realist analyses detailing causal mechanisms will be presented in forthcoming papers). Guided by Braun and Clarke's (36) reflexive thematic analysis approach, our process included: (1) data familiarization through reading and re-reading of transcripts; (2) inductively identifying key patterns and generating six initial codes (*conceptual and behavioral changes, mental health, peer and adult relationships, capacity, agency*, and *ripple effects*); (3) assigning data excerpts to the codes; (4) combining codes to construct potential themes informed our conceptual framework; (5) reviewing themes in relation to data; and (6) refining and finalizing theme names. In keeping with our convergent mixed-methods study design (19), qualitative and quantitative data were analyzed separately but in parallel as we interpreted the findings, presented below.

## Results

A total of 18 youth aged 15–17 participated in the Phase 1 Agenda Gap intervention across the two British Columbia cohorts. Of these participants, all 18 contributed to pre- and post-intervention surveys and post-intervention qualitative interviews. Youth participants predominantly identified being of non-white ethno-racial background (*n* = 17), while one youth identified as mixed descent (Indigenous and white). All the youth were currently attending high school (see Table 2 for additional demographic characteristics of youth participants). In addition to the demographic data collected through the survey, many of the youth self-identified as belonging to an equity-deserving group due to lived and living experience with mental ill health and associated health systems and services, being a recent immigrant or refugee, or being LGBTQ2+.

**Table 2:** Demographic characteristics of Agenda Gap youth participants.

**Youth participants**	***n =* 18**	%^*^
Gender Girl/Woman Boy/Man Non-binary	17 1 0	94 6 0
Age 15 16 17	5 8 5	28 44 28
Educational level Grade 9 Grade 10 Grade 11	5 4 9	28 22 50
Ethno-racial background Black (African, Afro-Caribbean, African Canadian decent) Southeast Asian (Vietnamese, Cambodian, Thai, Filipino, Indonesian, other Southeast Asian decent) East Asian (Chinese, Korean, Japanese, Taiwanese descent) Middle Eastern (Arab, Persian, West Asian descent (e.g., Afghan, Egyptian, Iranian, Lebanese, Turkish, Kurdish) Mixed descent	1 8 6 2 1	5 44 33 11 5

^*^% totals may not equal 100% due to rounding.

Four adult allies participated in a post-intervention qualitative interview. These participants had all engaged with Agenda Gap through their professional roles in the health (*n* = 1) and education sectors (*n* = 3) as policy and/or other decision makers. These results are visible in the theme area of community-level impacts.

### Pre- and post-intervention survey findings

Pre- and post-intervention knowledge assessments are presented to characterize understanding of core positive mental health concepts (mental health promotion literacy) pre- and post-intervention – (see Table 3). For most of the items (60%), a greater proportion of participants answered correctly at the post-intervention time point. However, for Mental Health Promotion there was no change in the proportion of participants answering the item correctly. With respect to Youth Policy Engagement, Youth Policy Strategies, and Intersecting Vulnerabilities, there was a 5.6% reduction in the proportion of participants answering correctly post-intervention. Overall, the average number of correct responses increased from 5.7 (SD 1.7) pre-intervention to 6.4 (SD 1.6) in the post-intervention assessment, however this difference was not statistically significant according to a paired samples *t*-test: *t*(16) = 1.04, *p* = 0.31.

**Table 3:** Description of pre- and post-intervention knowledge assessments.

	**Pre-test, *n =* 18, no. correct (%)**	**Post-test, *n =* 18, no. correct (%)**	**Improvement? Yes or no**
Mental health concept			
Resilience	2 (11.1)	8 (44.4)	Yes
Mental health	7 (38.9)	9 (50.0)	Yes
Contributors to mental health	15 (83.3)	16 (88.9)	Yes
Mental health promotion	2 (11.1)	2 (11.1)	No
Policy	10 (55.6)	12 (66.7)	Yes
Youth policy engagement	17 (94.4)	16 (88.9)	No
Youth policy strategies	13 (72.2)	12 (66.7)	No
Youth rights	9 (50.0)	11 (61.1)	Yes
Youth influence	16 (88.9)	18 (100)	Yes
Intersecting vulnerabilities	12 (66.7)	11 (61.1)	No
Total number correct Mean (SD)	5.7 (1.7)	6.4 (1.6)	Yes

Pre- and post-intervention group means and standard deviations are presented in Table 4 to characterize constructs of youth participants' positive mental health (i.e., self-efficacy, civic participation, resiliency, attachment, and critical consciousness). Reliability of the scales, assessed *via* Cronbach's alpha, ranged from 0.58–0.97. In general, there was a trend toward improved positive mental health from pre- to post-intervention assessment, apart from self-efficacy, resilience, and egalitarianism, which had similar average scores in pre- and post-intervention assessment. Paired samples t-tests using cases with complete data indicated a similar trend toward improvement over time. However, only scores on the Perceived Inequality scale were significantly different [General Self-Efficacy: *t*_(12)_ = −0.22, *p* = 0.83; Civic Participation: *t*_(13)_ = −1.67, *p* = 0.12; Resilience: *t*_(12)_ = −2.11, *p* = 0.06; Peer Support: *t*_(14)_ = −1.52, *p* = 0.15; Adult Support: *t*_(13)_ = −1.66, *p* = 0.12; Perceived Inequality: *t*_(14)_ = −2.26, *p* = 0.04; and Egalitarianism: *t*_(11)_ = 1.06, *p* = 0.31].

**Table 4:** Constructs of youth participants' positive mental health pre- and post-intervention.

	**Pre-intervention**			**Post-intervention**	
	**Reliability** ^*^	***M (SD)***	***n***	***M (SD)***	***n***
**Construct**					
General self-efficacy	0.65	3.28 (0.28)	17	3.28 (0.25)	15
Civic participation	0.88	3.29 (0.92)	17	3.57 (0.90)	16
Resilience	0.69	2.83 (0.21)	15	2.83 (0.19)	16
Peer attachment	0.58	4.55 (0.50)	17	4.63 (0.44)	17
Adult attachment	0.79	4.00 (0.90)	17	4.43 (0.44)	16
**Critical consciousness**					
Perceived inequality^**^	0.97	4.63 (1.27)	17	5.19 (0.92)	17
Egalitarianism	0.77	5.73 (0.42)	17	5.73 (0.47)	14

^*^Cronbach's Alpha assessed in pre-intervention survey; ^**^ significant at p < 0.05.

### Post-intervention qualitative interview findings

While the survey data provide indications of the impacts of Agenda Gap related to youth participants' knowledge and constructs of positive mental health, the interview data articulate the ways in which the intervention contributes to mental health promoting outcomes for youth and adult ally participants. These data also suggest ‘ripple effects' that hold the potential to address the determinants of mental health and equity across socioecological domains. Findings are organized thematically to illustrate perceived impacts at the individual through community levels, though, as described by participants, these impacts often span multiple domains. In presenting these themes below, illustrative participant quotes are used throughout to foreground youths' voices and expertise.

### Individual-level impacts: Personal transformation

#### Re-conceptualizing mental health

Across interviews, youth participants shared that Agenda Gap contributed to new and expanded understandings of mental health, as they were encouraged to connect program concepts and apply them to their everyday contexts. Specifically, youth identified the importance of recognizing mental health as a positive concept, distinct from mental illness. One youth related, “*This program helped me and a lot of others understand that mental health isn't mental illness.”* She went on to say, “*I understood how good mental health can look different for people…good mental health isn't just exercising or meditating, it can be the daily things in your life.”*

While this shift in understanding mental health was not well reflected in the knowledge assessment survey data, the qualitative data underscore substantial gains in youths' understanding and application of this new knowledge. Indeed, these new understandings extended youth participants' conceptualizations of mental health as an outcome of individual characteristics to also include the role of social and structural conditions, including positionality and inequities. One participant said she came to understand, “*how deep-rooted racism can affect mental health and how it's not just about personal change, it's more about community-based support.”* This new knowledge also extended their ideas about how mental health could be strengthened or promoted, including through policy change. One participant shared, “*I have a much better understanding of how policy affects me and how it can affect youth mental health*.” Even youth who came to the program with lived experience of the ways that mental health is impacted by social and structural determinants gained this understanding. One youth said, “*I knew beforehand how systems of oppression can impact mental health, that's something my family's experienced. But that policy change can help – that is not something I really considered much…”* This participant continued that Agenda Gap helped them to appreciate that “*…mental health isn't just dealt with in a therapist's office, but it can be dealt with through legislature as well.”*

As these youth participants' words so powerfully convey, and as is echoed in the quantitative results, there were pronounced shifts in mental health-related knowledge and critical consciousness as a result of Agenda Gap – providing new understandings that are informing how these youth think about and consider possible action to strengthen mental health.

#### Expanded social awareness and agency

Many participants expressed that their participation in Agenda Gap expanded their social awareness, increasing their sense of empathy and sensitivity to equity issues. According to one youth:

*It made me open minded in the sense that when I talk to other people or interact with others in my life, whether in school or with other youth, it makes me more like thinking in their shoes. To remember that not everyone thinks the way you do, not everyone has the same experiences or the same support systems that you do*.

This broadened awareness also extended to the social and structural determinants of mental health. As one youth described, Agenda Gap helped her to understand and respond to family dynamics impacting her mental health in new ways:

*No one talks about intergenerational mental health. And so, if my forefathers or foremothers went through something, now I'm going through and I can approach it differently. Agenda Gap taught me to use that upstream approach… It gave me that proactive way of looking at my mental health, which really helped me break some toxic cycles*.

This new way of looking at mental health was further described by other participants who explained that in addition to gaining awareness of the social and structural origins of mental health and illness, their participation in Agenda Gap contributed to a shift from feeling powerless to empowered and more equipped to take action:

…*How we could impact as youth, ‘cause a lot of youth, myself included, feel like nothing I say really matters cause it's all adults in charge. But actually realizing that we can change things and being able to present to [decision maker in the education system] was very empowering…*

This personal growth and desire to become engaged in social and political activities – or civic participation – was shared by other participants, who also described gains in self-efficacy that would help position them for success in this sphere. As one youth shared:

*Learning about all this shows that youth can do it. Like we're not just children that have to abide by the rules. We can be the rule makers or the rule changers. Learning about our rights, learning about our abilities, really boosted my confidence and I'm sure it boosted my group's confidence because we were instantly, like shaped into these leaders… You don't have to be old to be a leader or to create change. You can just voice your opinion and bring forward movements from here*.

Overall, there was a strong emphasis and enthusiasm among the youth participants about improvements in their sense of capacity for leading change to strengthen mental health and advance equity for themselves, their peers, and communities.

#### Implementing learnings in daily life

Many of the participants shared they incorporated the various skills learned and practiced during the Agenda Gap intervention into their everyday lives, with myriad benefits. For example, a number of youth said they developed communication and public speaking skills as a consequence of the interactive and youth-led design of the program. A participant who struggled with confidence in public speaking and asserting their voice before joining Agenda Gap observed, “*I definitely see a lot of changes in my peers. It's the same thing as me – we were all very quiet and then after our participation, we became more competent in speaking.” One* youth who led a community dialogue during the program said, “*I didn't know anything about dialogue coming in [to Agenda Gap] and I left with this new perspective on how to lead or hold a conversation in general. I found myself putting those techniques into use in my general life.”* She explained that as a result, “S*ome pretty hard conversations [outside Agenda Gap] went better than they could have because I used those techniques.”* Other youth shared that they experienced a growth in their confidence and communication skills, which in combination with their new and expanded conceptualization of mental health, motivated them to talk about the topic in settings where there had previously been stigma or other barriers, including amongst their friends, families, and sports teams.

Many participants also described adopting self-awareness and emotional regulation techniques that were presented and practiced during Agenda Gap sessions. Some of the youth were aware of or had tried these strategies in the past, but were skeptical because of previous experiences, found them difficult to do alone, or were unconvinced of their effectiveness. As one youth recounted:

*I always used to think like, oh, this [breathing exercise] is useless. Like, why do people do this? But then we actually did it properly. And we did it for multiple sessions. Eventually I got super used to it and I realized how helpful and how good it is..*.

Benefits of incorporating these strategies were described as including stress reduction, better quality sleep and improved mental health. One youth said:

*These activities were like a cherry on top, just to like help me with my personal mental health, especially with those meditation tactics and ways to boost self-confidence and being kind to yourself when you're going through a hard time and [to] not be so harsh with yourself*.

Similar to this participant, others also expressed growth in self-compassion as well as expanded personal coping strategies, which they articulated as a key benefit of participation in Agenda Gap.

#### Experiencing supportive community connections

Mirroring findings from the survey data capturing improvements in peer and adult attachment, youth participants shared that Agenda Gap contributed powerfully protective effects for mental health by engendering a sense of community connection that expanded their networks of social support and inspired further engagement. According to the youth, these community connections were derived from a sense of inclusion, safety, and ownership of the Agenda Gap process. Many participants described the role of safety and non-judgement in supporting them to be open and honest about what they were experiencing. As one participant shared, “*The most important aspect was for me the community that we created, because it was an extremely safe space and everyone could get as honest and just share as much as they wanted to.”*

For many participants, it was the first time they had experienced a validating, non-judgmental and empowering space, which inspired them to share the approach in other contexts. One participant expressed, “*there aren't many spaces where youth voices are valued or, even if they are there, they aren't accessible for all youth. Not all of us get to participate.... It's like* [typically] *reserved to the ‘special youth'.”* This sense of safety and validation within the context of strong adult and peer attachment was further attributed by some participants as having a positive impact on their mental health:

*It's the first place where I have adults or other students that I can openly talk to about my experiences or what is happening around us and not have to walk on eggshells, making sure what I said didn't offend anyone. Because all the time in this group, I felt supported. I felt validated… I didn't have people who would say these things before*.

She went on to say, “*having these people, having this space where I can openly talk about it was enough for me. And once I had this, I felt that I was generally becoming happier*.”

This sense of being “happier” – a key characteristic of positive mental health – was echoed by several other participants, who articulated the mental health promoting benefits of Agenda Gap participation. Youth participants also shared that their growing sense of connectedness had become a resource that created opportunities to expand their engagement with and support for their community:

*I have so many new contacts to talk to about this ‘cause before, it was just me, my therapist, and a few other friends that know a little more about mental health than the rest of the school. Now it's an entire network of allies. I can literally reach out to any single person I was working with at Agenda Gap and ask them for an idea if I'm doing a fundraiser or I can reach out to one of the school trustees who seemed really eager to help me*.

Opportunities for youth and adult allies to engage with one another supported youth to develop and use their voices to advocate for pathways for continued collaboration. According to youth participants, positive experiences with adult allies built trust that their efforts were worthwhile, and along with the confidence generated in the safety of the group, increased their motivation to engage and take action at a variety of levels. One participant articulated this sentiment in sharing:

*Because of my participation in Agenda Gap, I felt more motivated to actually go and comment and write stuff down and help out [in school initiatives]. I felt more competent in my knowledge of mental health and confident in my voice and knowing that what I'm sharing will probably get to someone…*

These strengthened relationships, and their related impacts, were a central feature of the youth participant interviews and provide nuanced insights into the role that peer and adult attachment plays in positive mental health through pathways of connectedness and belonging.

### Family-level impacts: Breaking down barriers to mental health through knowledge sharing

While the youth participants noted a variety of individual-level impacts because of their direct engagement with the intervention, they also described how their learnings were translating to shifts in their family's understandings of mental health and related dynamics and were also effective at disrupting entrenched and stigmatized beliefs. For example, one participant who described new confidence in her knowledge and right to voice her perspectives, shared how she was working to change conversations about mental health within her family. She explained, “*I feel like I've got a lot from Agenda Gap and I guess for me, my parents say, ‘oh, you have good ideas, I like what you shared with us, this really new and interesting!”'*

Similarly, another participant demonstrated her growing mental health promotion knowledge and described how she used this to broaden her mother's understandings of mental health to include an application of the impacts of social and structural determinants:

*I tried to explain the idea of intergenerational trauma to [my mom]. And I think she had questions. She didn't fully understand it. So, I tried my best to answer and she was “oh, that kind of made sense.” Not just that, but she, in fact, made a connection to our country back home. I had never heard a single adult in my life talk about the cycle of poverty. If I can explain this concept and my mom was able to understand it well enough to apply it to another situation that is definitely similar to this one, it's just, like, whoa! That's great because it was Agenda Gap. They gave me the tools to articulate my words and helped me explain what I meant*.

For other participants, generational norms and stigmatized beliefs about mental ill-health within their family contexts were described as a barrier to their own mental health. Agenda Gap was described as generating new understandings and language to talk about mental health in ways that impacted their family's knowledge. As one participant explained:

*My parents are, I wouldn't say they're old, but there's a generational gap obviously, and they don't understand [mental health] the way that I do. I don't expect them to fully understand it because there's kind of lack of education in their generation. So, I would talk about it… and they're starting to understand how it really is and how it really isn't. It's more than just mental illness, it's mental wellbeing. I would say they kind of understand it more now than they did before I joined*.

Indeed, the positive mental health orientation of mental health promotion was noted by some participants as providing an antidote to pervasive cultural stigma about mental health and illness – creating an entry point and the conditions for productive dialogue that is mental health supporting.

### Community-level impacts: De-problematizing youth and strengthening pathways to meaningful engagement

Beyond the individual- and family-level impacts, participation in Agenda Gap also created venues for youth to engage with allied adults and their broader social contexts. In doing so, it provided opportunities for community to observe youth expertise and gain insights about meaningful youth engagement and partnership. Both youth and adult participants indicated that this process shifted, and sometimes overturned, adult assumptions about youths' capacity to self-determine, voice their experiences, and meaningfully contribute to initiatives that improve conditions at a community level – a step toward changing the structures that create and maintain health and social inequities. For one healthcare decision maker participant, the experience motivated them to advocate for the meaningful engagement of youth, using a strengths-based approach, in their professional context:

*It really turned up the volume on my intention [to be] curious about the youth's experience and being curious about their strengths and really advocating strongly in meetings. I have really kind of recommitted to talking about the youth as doing the very best they can with the tools and structures that they have. How can we change the environment? How can we change their care team?... So that there's more accountability on the adults and less accountability, or not less accountability, but just different accountability, for the youth. [Engaging with Agenda Gap] was just such a good reminder of all the strengths and wisdom that youth bring*.

The words of this adult decision maker participant reflect deepened understandings of the critical nature of youth-adult attachment relationships to young peoples' wellbeing and the role that adult allies can play in facilitating meaningful, mental health promoting opportunities. This participant went on to share that their advocacy for youth expertise and engagement extended to the healthcare policy tables they attended:

*We've been talking a lot about policy level and program development and starting new teams in my area of practice. And this way of thinking I'd say, has been embedded in all of those. So, in some ways that's a tangible outcome or difference that like, as we're structuring who are we going to hire to build out new teams and how are we going to structure the policies and expectations of how those teams are going to function… I'd say my experience at the Agenda Gap workshop has tangibly informed my approach to those conversations*.

A teacher who joined Agenda Gap as an ally relayed that she was approached by a group of youth participants who came equipped to self-direct and advocate for themselves in their initiation of a mental health club at their school. Drawing on their Agenda Gap experience, the youth were able to maintain a strengths-based approach to mental health that ensured the sustainability and positive impact of the club and her involvement:

*I was a little bit worried about starting a club. I also didn't want it to be like group therapy. So, I think in seeing these students advocate for themselves… it's allowed me to kind of step back and say, ‘okay, you do you, and just kind of ask me what you want from me and I will provide that'. Whereas, I think at the beginning, I was really afraid that it would become – and it has happened in schools and I think this is why the other school had banned it – it can become kind of a negative space where people are reinforcing their own kind of mental health issues. So, I was really happy to see it didn't happen like that*.

In this way, the upstream and strengths-based understandings of mental health that youth participants gained through Agenda Gap were being translated to effect change within their broader social contexts and through their allyships. Youth participants also shared that they observed several other ripple effects – or community impacts. For example, one cohort worked with their teachers to create a presentation to the school district on strengthening its anti-racism policy. News of their efforts broadened support for their initiative and the school supported their request to start a Black, Indigenous, and People of Color (BIPOC) club. One youth related that she received more opportunities to lead events, while another shared that their teachers were more open to changing their instruction processes to support anti-racism objectives:

*After our presentations, a lot of our BIPOC teachers talked to other teachers and we also presented to them talking about the curriculum, the removal of the SLO (School Liaison Officer) [role], and how it affected a lot of students at [school]… We talked to the teachers about [engagement with the topic of slavery] and they changed their curriculum and changed their wording on the assignment*.

Another cohort directed their advocacy toward the need for spaces to support intergenerational dialogue about mental health to address ongoing stigma in their family and community contexts. These youth hosted a dialogue event by school and health authority decision makers and reported several positive impacts at various levels and across systems. Within the school setting, youth participants shared that teachers were more willing to directly address the topic of mental health. One youth said, “*I have teachers talk about mental health a lot. So, I think that's an outcome. They talk about it now in the lesson. They're like, ‘If you're struggling, I'm here for you, everyone has this, is struggling with this.”'* In one high school, the youth were also invited to provide a series of follow-up dialogues on an ongoing basis. One youth recounted, “*One of the district [leaders] who came to our dialogue, he actually proposed the idea of having a dialogue on one of our pro-D (professional development) days.”* At the school district level, a youth mental health advisory committee was approved and implemented, led by Agenda Gap youth alumni. Finally, at the broader community level, Agenda Gap youth were invited to deliver a dialogue for professionals involved with youth and youth mental health in their community.

Interest in continuing the Agenda Gap approach was referenced by youth and adult participants within and beyond the school setting, with a youth-serving health agency indicating their intention to continue with the dialogue model initiated by the youth. According to one youth participant:

*People are planning to make this not a one-term thing, but to continue implementing this kind of Agenda Gap in school and in the school community, as well. And more teachers are more aware of what the students are doing and they are more inspired of what we do, especially for health workers and social teachers. They are inspired to take this on as a next level*.

As this youth participant's words illustrate, there was great interest expressed by both youth and adult ally participants to continue to support and extend the initiatives that began as part of the Agenda Gap process. This bodes well for generating sustainable policy advocacy and change to continue the positive shifts that were initiated by the youth participants. In this way, the Agenda Gap is effectively positioned to continue to facilitate program impacts that span socioecological domains to strengthen the mental health and wellbeing of other youth and members of their broader communities.

## Discussion

Efforts to address and prevent mental ill health among youth have garnered widespread attention and growing investment in recent years, while a focus on strengthening positive mental health and wellbeing – the purview of mental health promotion – has remained more limited. Resultantly, there is a paucity of empirical evidence detailing the potential impacts achieved through adopting and implementing strengths-based and upstream mental health promotion initiatives. Drawing on mixed-methods data, this study offers important insights. Our findings, grounded in youth and adult ally perspectives, illustrate the promise and utility of mental health promotion *via* policy engagement and advocacy for generating positive mental health impacts across socioecological domains.

While further evaluation will be conducted in Phase 2, these Phase 1 findings emphasize the need for expanded investment in mental health programming explicitly guided by mental health promotion theory and principles. Many youth and adult ally participants articulated profound shifts in their understandings of mental health, moving from an illness-oriented, biomedical framing to one that now also includes an appreciation for, and application of, the social and structural determinants. Indeed, it is well recognized that the “drivers” of mental health and illness comprise the “complex interplay between neurobiological and psychosocial systems, risk and protective factors, and mental health systems and service utilization” (37). And yet, there continues to be limited investment in mental health promotion research, practice, and policy (3), effectively perpetuating narrow conceptualizations of mental health and, relatedly, intervention. With Agenda Gap, our study's qualitative findings suggest that the program's mental health promotion orientation supported youth to broaden their understandings of mental health and equity, while also contributing to gains in positive mental health for program participants. These expanded understandings of mental health were further accompanied by an appreciation for new opportunities and channels to strengthen mental health outcomes, particularly through policy advocacy. Policy advocacy as mental health intervention is responsive to the social and political nature of mental health and mental health inequities (6). While we are not suggesting that it is the sole means for promoting mental health, policy advocacy is positioned to influence beyond the health sector, to include other spheres shaping mental health and wellbeing, such as education, the environment, housing, justice and welfare (38). In this way, it provides a mechanism through which to create the social and structural conditions conducive to positive mental health. However, as we demonstrate in this study and as Knibbe et al. (39) also note, such policy advocacy ought to reflect diverse voices and expertise and acknowledge “issues of power and responsibility are at play” (p. 437). This is a salient consideration in the context of youth intervention, where power dynamics related to age, along with other social factors, have historically operated to exclude youth from policy and other decision-making processes (40), and where efforts to build skills and capacity remain limited (20). Relevant in considering the potential impacts of our intervention, youth engagement in social and political life is associated with a number of positive mental health outcomes, including greater peer and adult attachment, higher self-esteem, and stronger sense of identity (33).

Another key finding of this study centered on the opportunities that Agenda Gap created to demonstrate youth citizenship or capacity for “belonging, independence and equality, responsibility and participation, and shared existence and identity” (32). Youth have long been constructed within public discourses as a threat, in need of discipline and maturation before *earning* the right to have their needs heard and valued. As Hart (41) suggests, “This has led to a situation where young people are positioned as the passive recipients of citizenship policy rather than as active citizens in their own right. Indeed, in defining young people as not-yet-citizens they are, in effect, excluded not just from the formal rights of citizenship, but also from being treated with equality in terms of membership in society.” Supporting conditions for youth to voice their needs and influence the contexts and structures that impact their health and wellbeing is an area of growing interest, globally, and one that is enshrined as a basic human right within the Convention on the Rights of the Child (42), to which Canada, the setting for the Agenda Gap intervention, is a signatory (43). Importantly, youth and adult ally participants expressed that Agenda Gap shifted adult perceptions about youths' capacity for self-determination and citizenship and led to plans for continued engagement in social and political life.

Additionally, our study provides much-needed evidence on the processes and impacts of adopting a socioecological approach to mental health promotion intervention. Much of the mental health promotion literature describes programming targeted at changing intrapersonal behavior (14), though the full potential of this orientation is best achieved by leveraging its socioecological emphasis (44). As youth in our study emphasized, such intervention can benefit when it is responsive to the multi-level social and structural factors shaping access to determinants of good mental health and wellbeing as well as equity. Indeed, without the consistent adoption of a socioecological model, mental health promotion intervention risks operating to maintain the dominant, yet insufficient, conceptualization of wellbeing as an individual-level experience or state of mind, rather than as a collective or socially mediated phenomena (45). This narrower view has fit conveniently within the neoliberal political landscape that has characterized much of North America and Europe for the past several decades, responsibilizing the public for their mental health outcomes, while ignoring issues of inequity and injustice. As Knifton (45) aptly questions, “Why are we getting people to reframe their social situation without changing peoples' social situations?” Socioecologically oriented mental health promotion holds great promise for disrupting the status quo. We argue that this disruption is overdue and required to advance intervention that is intentionally designed to address the broad spectrum of factors that shape mental health, from individual behaviors and practices through to societal conditions and issues of (in)justice.

While socioecological mental health intervention represents an important path to pursue, it is not without its challenges. It is a complex undertaking and requires thoughtful development of aligned measures and metrics to monitor effect. This challenge is not unique to mental health promotion. It is a struggle shared by scholars within the broader public and population health field, where demonstrating the impact of community-based, multi-level (particularly structural) intervention is a priority methodological pursuit (46). Moreover, and specific to mental health promotion, many measures of positive mental health and wellbeing remain underdeveloped or un-validated, as was the case with several of the measures adopted for the present study (e.g., peer and adult attachment, civic engagement). An additional challenge with currently available measures of positive mental health is that many do not have sufficient item discrimination, that is, the items are framed in a way that produces little variability in scores across participants (47). This was an issue in our study alongside ceiling effects, wherein the overall group of participants scored high on measures of positive mental health at baseline, leaving little room to demonstrate improvement over time. Despite this, the quantitative results remain useful in providing information on the utility of various scales for future use in Agenda Gap research and evaluation in Phase 2 and beyond, as well as in research on positive mental health more broadly. Indeed, in light of our data, we argue there is a need for further identification, development and validation of scales that exhibit sensitivity and responsiveness to change (48) and can distinguish reliably between higher and lower levels of the underlying positive mental health constructs. Guided by these Phase 1 findings, careful consideration of additional or alternative mental health measures will be trialed alongside future iterations of Agenda Gap.

While this study makes important contributions to guide the science and practice of mental health promotion intervention, there are limitations to acknowledge. The data presented represent the perspectives and experiences of youth from one provincial region of Canada. While these participants were intentionally diverse in their social positions, identities, and lived experiences, future research will benefit from the inclusion of youth from other geographical contexts to confirm the transferability of findings. Furthermore, while we argue that previous research, and our results here, support the notion that youth-led policy engagement and advocacy can yield mental health benefits, we acknowledge that such efforts could take multiple forms. More broadly, while a focus on social justice and addressing health and social inequities through the social determinants of mental health has deep roots in Canadian public health (49), mental health promotion intervention could be informed by theoretical perspectives beyond those adopted and utilized in our intervention. As such, youth cohorts are encouraged to steer each iteration of the intervention and policy foci identified. Furthermore, while efforts were made to involve youth who have previously been excluded from opportunity, including through partnered recruitment methods and the provision of honoraria, barriers to participation likely remain; in some global contexts, for example, there may be risks to youth who engage in policy advocacy. However, we would suggest that such realities ultimately serve as an argument for bringing *greater* focus to interventions that address wider social and structural determinants of mental health.

Additionally, while the mixed methods design of this study provides rich and nuanced insights into participants' perceptions of Agenda Gap impacts, this was a Phase 1, exploratory study and was underpowered to support quantitative analyses beyond those presented. This was compounded by the presence of ceiling effects for many of the measures used. As Agenda Gap is refined and tested with additional groups of youth in Phase 2 activities, the sample sizes will grow and it is anticipated that this will permit realist analyses to explore the causal mechanisms of program effects, including how these may vary by participant characteristics and context (23).

Mental health promotion – particularly that which adopts a socioecological orientation – holds untapped potential for strengthening positive mental health and wellbeing for youth, as well as their families, communities and society at large. Yet research, policy and practice in this field remains under resourced, with the burden for this work largely falling to individual schools and community organizations. To leverage the full potential of this approach, there is a need for a radical shift in the ways in which mental health is conceptualized (i.e., acknowledging the social and structural origins of mental health and illness, individually and collectively). This must be accompanied by bold actions by government and other decision makers to commit to realizing a population approach to mental health, inclusive of promotion, alongside prevention, treatment and recovery. It will also require ongoing efforts to challenge prominent (problematic) beliefs about youth capacity and to create spaces where their voices and perspectives are actively sought, valued and actioned. Without such efforts, solutions to youth mental health will remain illness-centric and reactionary, failing to progress in attending to the upstream determinants of good mental health and equity.

## Data availability statement

The raw data supporting the conclusions of this article will be made available by the authors, without undue reservation.

## Ethics statement

The studies involving human participants were reviewed and approved by the University of British Columbia Behavioural Research Ethics Board (H17-001602). Written informed consent from the participants' legal guardian/next of kin was not required to participate in this study in accordance with the national legislation and the institutional requirements.

## Author contributions

EJ and RH-S led conceptualization of the study. EJ directed project administration, formal analysis, and writing – original draft. LM, ZD, and CR contributed to analysis and writing – original draft. CM, TH, TG, CL, SB, RR, and RH-S contributed to analysis and writing – review and editing of manuscript. All authors contributed to the article and approved the submitted version.
